# Late Presentation of Oral Chronic Graft Versus Host Disease Manifesting As Hyperkeratotic Plaque: A Case Report

**DOI:** 10.7759/cureus.60147

**Published:** 2024-05-12

**Authors:** Mutaz F Felemban, Rasha S AlRasheed, Rana S Alshagroud, Abdullah M Aldosari

**Affiliations:** 1 Oral Medicine and Diagnostic Sciences, King Saud University, Riyadh, SAU

**Keywords:** opmd, cgvhd, oral potentially malignant disorder, oral chronic graft-versus-host disease, oral graft-versus-host disease

## Abstract

Hematopoietic stem cell transplantation is the only curative intervention for myelodysplastic syndrome, with graft-versus-host disease (GVHD) being a frequently encountered consequence. GVHD is classified as acute (aGVHD) or chronic (cGVHD). The oral cavity is the most impacted by chronic. Oral manifestations of cGVHD are variable and include plaque, Wickham striae, and lichenoid patches. In order to prevent malignant misdiagnosis, the 2014 NIH consensus report decided to exclude white plaque as a diagnostic indicator for oral cGVHD. Nevertheless, it is still possible to classify a white plaque lesion as cGVHD through histological confirmation. The performance of a biopsy should be undertaken following meticulous consideration and a thorough evaluation of the associated risks and benefits. The in-depth review of oral cancer risk assessment is crucial, necessitating a careful review of multiple factors to accurately estimate the likelihood of malignant transformation in individuals with oral cGVHD. This report describes a case of oral cGVHD manifesting as hyperkeratotic plaque lesions confirmed by histopathology in a 62-year-old man who received an allogeneic hematopoietic stem cell transplant over a decade ago.

## Introduction

Myelodysplastic syndromes (MDS) comprise a group of malignancies of the bone marrow characterized by abnormal cellular development, persistent cytopenia, and the potential for progression to acute myeloid leukemia [1-3]. These conditions arise due to genetic alteration in hematopoietic stem cells. The disease initiates as a clonal process involving transformed hematopoietic stem and progenitor cells that gradually acquire various oncogenic mutations and cytogenetic abnormalities. These anomalies can be present at diagnosis or develop during the disease, leading to dysplasia and ineffective hematopoiesis [4-7]. The gradual loss of differentiation and maturation results in impaired functioning of blood cells, particularly platelets and granulocytes [8].

The molecular cytogenetic analysis classifies MDS into several subtypes, including MDS with deletion (5q), MDS with SF3B1 gene mutation, and MDS with bi-allelic TP53 alterations [9,10]. Deletion of the long arm of chromosome 5, known as del(5q), represents a unique subtype of MDS and is one of the recurring chromosomal abnormalities observed in MDS cases, accounting for approximately 10%-15% of all cases. Currently, allogeneic hematopoietic stem cell transplantation (HSCT) is the only therapeutic option that offers the potential for a cure [11,12].

Regrettably, the use of allogeneic HSCT often leads to graft-versus-host disease (GVHD), which is an inflammatory syndrome where the transplanted cells attack the recipient's tissues [13]. GVHD can be categorized into two main types, acute (aGVHD) and chronic (cGVHD), which differ in how they develop, when symptoms appear, and their clinical manifestations. Although aGVHD typically has a limited duration, cGVHD can persist for several years, necessitating long-term immunosuppressive therapies and exposing patients to various late complications [14].

The oral cavity is commonly affected by cGVHD. The buccal mucosa and lateral sides of the tongue are the most frequently involved areas [15]. Oral manifestations can include lichenoid mucosal disease, salivary gland dysfunction, and sclerotic changes. The mucosal changes are usually characterized by white striae, erythema, and ulcers that cause sensitivity to spicy, acidic, and strongly flavored foods and drinks [16]. In gingival tissues, cGVHD can cause desquamation, erythema, and sporadic color changes. The sensitivity of the gingiva can impede proper oral hygiene and contribute to plaque-induced gingivitis [15].

There has been scant attention given in the literature to describe the specifics of the oral presentation of cGVHD. The manifestations can vary in severity and clinical presentation. The manifestations can include Wickham striation, erythema, and plaque formation. However, the NIH group revised the diagnostic criteria in 2014 to recommend removing hyperkeratotic plaques as an oral manifestation of cGVHD. Oral cGVHD is considered a risk factor for developing oral squamous cell carcinoma (OSCC) and is included as an oral potentially malignant disorder (OPMD) [17]. This report describes a case of oral cGVHD presenting a decade after receiving the allogeneic hematopoietic stem cell transplantation as hyperkeratotic plaques confirmed by histopathology. 

## Case presentation

A 62-year-old male patient with a history of diabetes, dyslipidemia, and vitamin D deficiency controlled by metformin, simvastatin, and vitamin D supplements was diagnosed with MDS 5q deletion in 2005. Following this, he underwent allogeneic hematopoietic stem cell transplantation in 2007 and received a two-month course of 50 mg cyclosporin for graft versus host disease prophylaxis. In June 2023, the patient presented to the oral medicine clinic at King Saud University Dental Hospital to address a concern about an asymptomatic white lesion on the buccal mucosa, which had developed three months prior without any identifiable predisposing factors.

**Figure 1:**
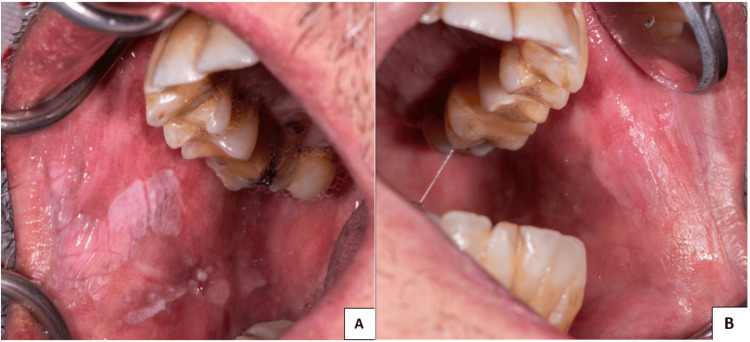
Right buccal mucosa and left buccal mucosa. (A, B) Oral changes with well-demarcated hyperkeratotic plaques on the right and left buccal mucosa.

Upon examination, thickened multiple white plaques are seen on the right and left buccal mucosa. The lesions are characterized by a well-defined border, variable rough surfaces, and a firm texture, and are not scraped off (Figures 1A, 1B).

**Figure 2:**
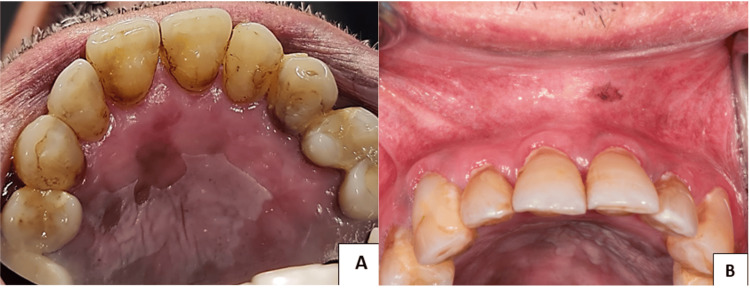
Anterior hard palate and upper labial vestibule. (A) Homogenous well-demarcated white lesion with hyperpigmentation on the hard palate. (B) Solitary well-demarcated brown pigmentation on the upper labial vestibule.

**Figure 3:**
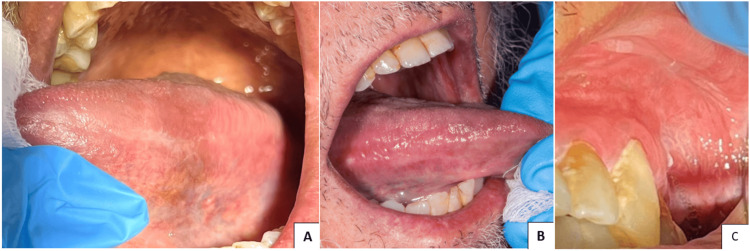
Left and right lateral borders of the tongue and upper left labial vestibule. (A-C) Radiating whitish-gray lines on the right and left lateral borders of the tongue and upper right labial vestibule.

Furthermore, a distinct smooth white patch has been identified on the hard palate. This lesion displays well-defined boundaries and a firm texture that cannot be removed. Moreover, brown pigmentation was observed on both the hard palate and the upper labial mucosa. (Figures 2A, 2B). Also, there were noticeably radiating whitish-gray lines on the tongue's right and left lateral borders and upper right labial vestibule (Figures 3A-3C).

**Figure 4:**
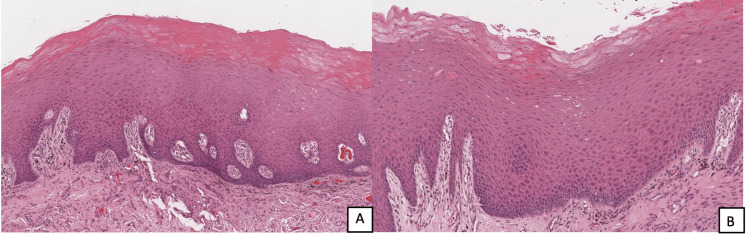
Photomicrographs of the specimen from the right buccal mucosa. (A) Epithelial hyperkeratosis and acanthosis with the mild lichenoid lymphocytic infiltrate. H&E, x40. (B) Higher power showing epithelial dyskeratotic cell and melanin incontinence associated with melanophages. H&E, x100.

The preliminary graft versus host disease diagnosis was proposed based on the patient's medical history and clinical examination. An incisional biopsy was obtained from the right buccal mucosa to verify this diagnosis and obtain histopathological confirmation. Microscopic examination of the specimen revealed a wedge of oral mucosa surfaced by nonkeratinized stratified squamous epithelium with surface hyperkeratosis, acanthosis, and scattered apoptosis. Interestingly, only one large dyskeratotic cell was observed in the present case. Rete ridges are either short and pointed or long and rounded. A mild band of lymphocytic infiltrate associated with a band of fibrosis is present in the superficial lamina propria. In addition, mild melanocytic hyperplasia associated with basal cell hyper-melanosis, melanophages, and melanin incontinence are noted (Figures 4A, 4B). All together, these features with the clinical presentation are consistent with the diagnosis of cGVHD. As the lesions are entirely asymptomatic, no medication is prescribed, close follow-up is planned, and a referral to oncology department is considered.

## Discussion

GVHD is a post-transplant complication characterized by the acute or chronic development of an autoimmune-like condition seen between 50% and 80% of individuals who underwent allogeneic transplantation [18].

Oral cGVHD typically manifests within the initial three years following HSCT and can exhibit either localized organ-specific involvement or diffuse systemic involvement, impacting multiple regions of the body [19]. A total of eight cases of cGVHD have been reported in a study conducted by Estela de la Rosa García et al. n in which all patients exhibited the emergence of lesions within a timeframe ranging from 117 to 507 days post-transplantation [20]. Furthermore, in a recent case series of six patients with oral cGVHD, all of whom were diagnosed with cGVHD within the first year of their HSCT [21]. The oral lesion in the presented case had a noteworthy development period of more than a decade.

Individuals with GVHD usually experience mild pain while at rest; oral mucosal thinning and ulceration frequently result in increased pain sensitivity. The symptoms may be aggravated by acidic, spicy, or highly flavored foods, carbonated beverages, and alcohol or alcohol-derived products. It is essential to emphasize that most individuals exhibit symptoms [22,23]. However, in our case, the patient exhibits no symptoms at rest, and even after exposure to known triggers that frequently induce symptoms.

The diagnosis of oral GVHD relies on a visual examination, which is further supported by the patient's medical history and clinical presentation. The presence of lichen planus-like alterations is indicative of the condition and does not necessitate the need for a confirmatory biopsy. The oral clinical manifestations of this condition can manifest as Wickham striae, lichenoid patches, or plaque [24]. However, the most current update on the NIH consensus report in 2014 eliminated the inclusion of white plaque as a diagnostic sign of oral chronic graft-versus-host disease (cGVHD) to prevent the misdiagnosis of malignant transformation. It is imperative to acknowledge that a white plaque lesion can be diagnosed as chronic graft-versus-host disease (cGVHD) with histological confirmation [21].

Moreover, it is crucial to differentiate between chronic graft-versus-host disease (cGVHD) and idiopathic leukoplakia due to the heightened susceptibility to oral malignancy following HSCT [25,26]. In addition, it is important to distinguish lichen-like alterations from alternative factors that might lead to oral hyperkeratosis, such as reactive reasons (frictional or chemical-induced) or infectious causes (pseudomembranous and hyperplastic candidiasis). In the case we report, the diagnosis of the plaque lesions was verified with histopathology, and the possibility of other lesions was excluded by obtaining a thorough history and careful clinical examination.

Since the suggestion of the minimal criteria for histopathological diagnosis of oral cGVHD by NIH group consensus, it continues to be a subject of study. The current 2014 criteria include leukocyte exocytosis, keratinocyte apoptosis, and lichenoid interface lymphocytes [27]. In this case report, the histopathological examination did reveal scattered apoptosis, one large dyskeratotic cell, and mild lichenoid interface inflammation. In addition, fibrosis of the stroma was noted with no evidence of minor salivary glands. In such complex disease processes, clinical correlation is highly crucial as histopathology represents only a snapshot of the actual pathologic process. 

Oral cGVHD poses significant risks for the development of OSCC [17]. The International Bone Marrow Transplant Registry in 1997 revealed that there is a relative risk of 6.0 associated with oral cGVHD in the formation of oral SCC following HSCT.

Moreover, 50% of invasive carcinomas manifested as white plaques in a group of cGVHD patients who had dysplastic or malignant oral lesions. However, this subset of cGVHD patients with white plaques does not reflect all cGVHD patients with white plaques. Therefore, the risk of malignant transformation of white plaques in cGVHD patients remains unknown [25].

The case series by Pukhalskaya et al. reported six cases of oral cGVHD manifesting as white plaques, with only one case demonstrating OSCC. In that series, the authors emphasized that despite the exclusion of “white plaque” from the criteria of oral cGVHD, the disease can still have this presentation even without lichen planus-like changes [21].

Since most patients with GVHD are immunocompromised, the decision to obtain a biopsy should be made after careful consideration. A biopsy may have adverse effects that outweigh the benefits of the procedure relative to its risks, such as bone infection, neurological deficits, chronic pain, and diminished quality of life. In addition, repeated biopsies may result in scar tissue formation, which may make subsequent follow-ups and clinical evaluation of the lesion challenging.

In addition, it is essential to carefully consider risk factors when estimating the risk of malignant transformation in patients with oral cGVHD. Tobacco use and alcohol consumption have been associated with OSCC. Also, numerous risk factors, such as family history, local chronic trauma, radiation therapy, and an antioxidant-deficient diet, can be associated with OCSS [27]. Our patient's risk factors were thoroughly evaluated, and fortunately, he is considered to have a low risk of developing OSCC. The patient was instructed to maintain a healthy diet and periodic follow-up was planned to identify any suspicious lesions to be biopsied.

## Conclusions

The present study describes a case of oral cGVHD characterized by the presence of hyperkeratotic plaques, which were further validated through histopathological examination. Based on the 2014 NIH consensus study, white plaque is no longer considered a diagnostic feature for oral cGVHD. Although, it remains feasible to categorize a white plaque lesion as cGVHD by histological confirmation, however, the execution of a biopsy procedure necessitates careful deliberation and a comprehensive assessment of the potential risks and benefits. The comprehensive evaluation of the risk of oral cancer is of utmost importance, requiring a meticulous examination of several risk factors to precisely determine the probability of malignant progression in individuals affected by oral cGVHD and periodic follow-up should be planned to biopsy any clinically specious lesions.
